# Supplementary material for: A low-cost paper-based synthetic biology platform for analyzing gut microbiota and host biomarkers
Source: Nat Commun. 2018 Aug 21;9:3347. doi: 10.1038/s41467-018-05864-4 (PMC6104080; doi:10.1038/s41467-018-05864-4)
Supplement: Supplementary file 1 — Supplementary Information [file 41467_2018_5864_MOESM1_ESM.pdf]

Supplementary Information for

A low-cost paper-based synthetic biology platform for analyzing gut microbiota and host biomarkers

Takahashi et al.

Table of contents:

	Description	Page
Supplementary Table 1	Important DNA sequences	2
Supplementary Table 2	16S sensor and trigger sequences	3
Supplementary Table 3	Species specific sensor and trigger sequences	4
Supplementary Table 4	16S NASBA primers	5
Supplementary Table 5	Species specific NASBA primers	6
Supplementary Table 6	Host biomarker sensor and trigger sequences	7
Supplementary Table 7	Host biomarker NASBA primers	8
Supplementary Table 8	qPCR primers and probes	9
Supplementary Table 9	Targeted species specific gene regions	10
Supplementary Table 10	Host biomarker mRNA standard sequences	12
Supplementary Table 11	qPCR performance characteristics	13
Supplementary Figure 1	16S toehold switch sensor screen	14
Supplementary Figure 2	Chemical structure probing data for *E. coli* 16S NASBA primers	15
Supplementary Figure 3	16S sensor orthogonality data	16
Supplementary Figure 4	*C. difficile* 16S sensor alignment	17
Supplementary Figure 5	Species-specific toehold switch sensor function	19
Supplementary Figure 6	Species-specific sensor orthogonality data	20
Supplementary Figure 7	Mathematical model of NASBA process	21
Supplementary Figure 8	NASBA time courses	22
Supplementary Figure 9	Species-specific bacterial calibration curves	23
Supplementary Figure 10	mRNA detection in stool RNA background	24
Supplementary Figure 11	Quantification of species-specific mRNAs in clinical samples	25
Supplementary Figure 12	Host biomarker sensor and NASBA validation	26
Supplementary Figure 13	Host biomarker calibration curves	27
Supplementary Figure 14	*C. difficile* toxin sensor and NASBA validation	28
Supplementary Table 12	*C. difficile* toxin DNA pcr results	29
Supplementary Figure 15	Correlation of *E. coli* species specific mRNA to cell count	30
Supplementary Figure 16	Demonstration of platform using an in-house cell-free system	31
Supplementary Figure 17	Performance of NASBA reactions using individually mixed components	32
Supplementary Note 1	Toehold switch sensor design script	33
Supplementary Note 2	Description of NASBA mathematical model	34

Supplementary Table 1: Important DNA sequences

	**Sequence**
T7 promoter	taatacgactcactatagg
T7 terminator	ctagcataaccccttggggcctctaaacgggtcttgaggggttttttg
Linker	aacctggcggcagcgcaaaag
GFPmut3b	atgcgtaaaggagaagaacttttcactggagttgtcccaattcttgttgaattagatggtgatgttaatgggcacaaattttctgtcagtggagagggtgaaggtgatgcaacatacggaaaacttacccttaaatttatttgcactactggaaaactacctgttccgtggccaacacttgtcactactttcggttatggtgttcaatgctttgcgagatacccagatcacatgaaacagcatgactttttcaagagtgccatgcccgaaggttacgtacaggaaagaactatatttttcaaagatgacgggaactacaagacacgtgctgaagtcaagtttgaaggtgatacccttgttaatagaatcgagttaaaaggtattgattttaaagaagatggaaacattcttggacacaaattggaatacaactataactcacacaatgtatacatcatggcagacaaacaaaagaatggaatcaaagttaacttcaaaattagacacaacattgaagatggaagcgttcaactagcagaccattatcaacaaaatactccgattggcgatggccctgtccttttaccagacaaccattacctgtccacacaatctgccctttcgaaagatcccaacgaaaagagagaccacatggtccttcttgagtttgtaaccgctgctgggattacacatggcatggatgaactatacaaa

Supplementary Table 2: 16S sensor and trigger sequences

Bacteria	Addgene ID	Sensor sequence	Trigger sequence
B. fragilis	110696	GCTTATTCATATAATACATACAAAACA GTATACATACGGACTTTAGAACAGAG GAGATAAAGATGGTATGTATACTG	GTATGTATACTG TTTTGTATGTATT ATATGAATAAG
B. thetaiotaomicron	110697	GCAAAATTCCACACGTGGAAAACTTT ATTCCCATATAGGACTTTAGAACAGA GGAGATAAAGATGTATATGGGAATT	TATATGGGAATA AAGTTTTCCACG TGTGGAATTTTG
E. coli	110698	GAACGTCAATGAGCAAAGGTATTAAC TTTACTCCCTTGGACTTTAGAACAGA GGAGATAAAGATGAAGGGAGTAAAG	AAGGGAGTAAAG TTAATACCTTTGC TCATTGACGTT
B. longum	110699	GGGTGCTTATTCAACGGGTAAACTCA CTCTCGCTTGCGGACTTTAGAACAGA GGAGATAAAGATGGCAAGCGAGAGC	GCAAGCGAGAGT GAGTTTACCCGT TGAATAAGCACC
B. adolescentis	110700	GCGAAGGGCTTGCTCCCAGTCAAAA GCGGTTTACAACGGACTTTAGAACAG AGGAGATAAAGATGGTTGTAAACCGA	GTTGTAAACCGC TTTTGACTGGGA GCAAGCCCTTCG
B. breve	110701	GAAGTGCCTTGCTCCCTAACAAAAGA GGTTTACAACCGGACTTTAGAACAGA GGAGATAAAGATGGGTTGTAAACCA	GGTTGTAAACCT CTTTTGTTAGGG AGCAAGGCACTT
E. rectale	110702	GTCATTATCTTCCCTGCTGATAGAGC TTTACATACCGGGACTTTAGAACAGA GGAGATAAAGATGCGGTATGTAAAC	CGGTATGTAAAG CTCTATCAGCAG GGAAGATAATGA
R. hominis	110703	GCATTCTTCTTCCCTGCTGATAGAGC TTTACATACCGGGACTTTAGAACAGA GGAGATAAAGATGCGGTATGTAAAT	CGGTATGTAAAG CTCTATCAGCAG GGAAGAAGAATG
C. difficile	110704	GGCCGGGGCTTCCTCCTCAAGTACC GTCATTATCTTCGGACTTTAGAACAG AGGAGATAAAGATGGAAGATAATGAC	GAAGATAATGAC GGTACTTGAGGA GGAAGCCCCGG C
F. prausnitzii	110705	GCGTCATTATCTTCCTCAACAACAGG AGTTTACAATCGGACTTTAGAACAGA GGAGATAAAGATGGATTGTAAACTA	GATTGTAAACTC CTGTTGTTGAGG AAGATAATGACG

Supplementary Table 3: Species-specific sensor and trigger sequences

Bacteria	Addgene ID	Sensor sequence	Trigger sequence
B. fragilis	110706	GATATCTTCTGCTTCCTGAATTATTATTGGGTCCGTTGGACTTTAGAACAGAGGAGATAAAGATGAACGGACCCAAG	AACGGACCCAATAATAATTCAGGAAGCAGAAGATAT
B. thetaiotaomicron	110707	GTTACTGTATGACCGGCTTTGTGTGGTGTTGCTCCTTGGACTTTAGAACAGAGGAGATAAAGATGAAGGAGCAACAC	AAGGAGCAACACCACACAAAGCCGGTCATACAGTAA
E. coli	110708	GCGGTTCAAACGTGGAAACGAAATAGATATCCGTCTTGGACTTTAGAACAGAGGAGATAAAGATGAAGACGGATATC	AAGACGGATATCTATTTCGTTTCCACGTTTGAACCG
B. longum	110709	GATATCTTCTGCTTCCTGAATTATTATTGGGTCCGTTGGACTTTAGAACAGAGGAGATAAAGATGAACGGACCCAAG	GTAGATAAGGCGCTGAACATTGATGCACAAGAACCT
B. adolescentis	110710	GGCAAACAAAGGCGCATAAGGAAGGAAATACCTGAATGGACTTTAGAACAGAGGAGATAAAGATGATTCAGGTATTA	ATTCAGGTATTTCCTTCCTTATGCGCCTTTGTTTGC
B. breve	110711	GTAACCGCCTGATTGAACGCGGCAATGTCAGTCCTGCGGACTTTAGAACAGAGGAGATAAAGATGGCAGGACTGACA	GCAGGACTGACATTGCCGCGTTCAATCAGGCGGTTA
E. rectale	110712	GGAAGGAAATGATGAGTCAAACGGTATCTTTACAATAGGACTTTAGAACAGAGGAGATAAAGATGTATTGTAAAGAG	TATTGTAAAGATACCGTTTGACTCATCATTTCCTTC
R. hominis	110713	GAGCTTGTCCATATCCGCCTTCTCAAAGCTCTTCTCGGGACTTTAGAACAGAGGAGATAAAGATGCGAGAAGAGCTC	CGAGAAGAGCTTTGAGAAGGCGGATATGGACAAGCT
C. difficile	110714	GCCATTATAGCCATTCCACTTATACATGCAATTGTTCGGACTTTAGAACAGAGGAGATAAAGATGGAACAATTGCAG	GAACAATTGCATGTATAAGTGGAATGGCTATAATGG
C. difficile toxin B	110715	GTGTTTTTTCAGTTTCTACAAATATAGATTCTCCAGTGGACTTTAGAACAGAGGAGATAAAGATGACTGGAGAATCT	ACTGGAGAATCTATATTTGTAGAAACTGAAAAAACA
F. prausnitzii	110717	GAAACGCACAGCAGCCCAAACTTCACAGGGTTTGACCGGACTTTAGAACAGAGGAGATAAAGATGGGTCAAACCCTT	GGTCAAACCCTGTGAAGTTTGGGCTGCTGTGCGTTT

Supplementary Table 4: 16S NASBA primers

Bacteria	Forward primer	Reverse primer
B. fragilis	aattctaatacgactcactatagggagaaggGGATGAAGGCTCTATGGGTCGT	TATTACCGCGGCTGCTGGCA
B. thetaiotaomicron	aattctaatacgactcactatagggagaaggAAGGATGACTGCCCTATGGGTT	ATCCTTATTCATATGGTACATA
E. coli	aattctaatacgactcactatagggagaaggTATGAAGAAGGCCTTCGGGTT	GTGCTTCTTCTGCGGGTAACGT
B. longum	aattctaatacgactcactatagggagaaggAGGGATGGAGGCCTTCGGGT	AGCCGGTGCTTATTCAACGGGTAA
B. adolescentis	aattctaatacgactcactatagggagaaggATGACGGCCTTCGGGTTGTAAACC	TATTACCGCGGCTGCTGGCA
B. breve	aattctaatacgactcactatagggagaaggAGGGATGGAGGCCTTCGGGT	TATTACCGCGGCTGCTGGCA
E. rectale	aattctaatacgactcactatagggagaaggAGCGAAGAAGTATTTCGGTAT	GTGCTTCTTAGTCAGGTACCGT
R. hominis	aattctaatacgactcactatagggagaaggAGAAGTATTTCGGTATGTAAAGCT	TCACATCAGACTTGCCGTACCG
C. difficile	aattctaatacgactcactatagggagaaggACTCTGTCCTCAAGGAAGATAATGA	TATTACCGCGGCTGCTGGCA
F. prausnitzii	aattctaatacgactcactatagggagaaggCGTGGAGGAAGAAGGTCTTCGGAT	AGTAATTCCGGACAACGCTTGT

Supplementary Table 5: Species-specific NASBA primers

Bacteria	Forward primer	Reverse primer
B. fragilis	aattctaatacgactcactatagggagagaaTTCAGATCGTAACGGACCCAAT	TCCTGTCCCAGTGATGATTTCT
B. thetaiotaomicron	aattctaatacgactcactatagggagaaggCCGACTTCGGAACGCTTATAGA	TGAAACGTATGCGGTAGCTGAA
E. coli	aattctaatacgactcactatagggagaaggCAAACTACGACGTCATCATTTAGC	GTTGACGGTTCAAACGTGGAAA
B. longum	aattctaatacgactcactatagggagaaggAGCTCGCTGATGGCAGTGTGGTA	TTTCTCCCGCAATAACGTTGAAG
B. adolescentis	aattctaatacgactcactatagggagaaggTTTACCTTGCCTTGTGACTGCAATA	CCGATTCCAACATTACGAATGCAAA
B. breve	aattctaatacgactcactatagggagaagaGGAAGCCGAACCTTGAAACG	AGCGGTGCAGTATGGGCGTA
E. rectale	aattctaatacgactcactatagggagaaggGTCGACAGATTGAGACCATGTCA	TAACGTGTGTCCCGAAGGAAAT
R. hominis	aattctaatacgactcactatagggagaaggGAGCGAATGAGAAGACGTTGGA	CGTGCGTTGTAAACAGTGACTT
C. difficile	aattctaatacgactcactatagggagaaggAATGCTGATAGGGTGTTGCGTT	TCAAGCATACACAATATTACCATTATAGCCA
C. difficile toxin B	aattctaatacgactcactatagggagaagAGATTCTCATTTTATATCTTTTGAGGACAT	ATTAGCATATTCAGAGAATATTGTTTTTTCAG
F. prausnitzii	aattctaatacgactcactatagggagaagaTGACTTGCGGCTATAATAGAAACG	TATTCCAAAACGCACAGCAGCCCA

Supplementary Table 6. Host biomarker sensor and trigger sequences

Gene	Addgene ID	Sensor sequence	Trigger sequence
Calprotectin *S100A9*	110717	GTGGTGGAAGGTGTTGATGATGGTCT CTATGTTGCGTGGACTTTAGAACAGA GGAGATAAAGATGACGCAACATAGT	ACGCAACATAGAG ACCATCATCAACA CCTTCCACCA
CXCL5	111907	GCATGCGTGCTCATTTCTCTTAATCA GTTTTCCTTGTGGACTTTAGAACAGA GGAGATAAAGATGACAAGGAAAACA	ACAAGGAAAACTG ATTAAGAGAAATG AGCACGCATG
IL-8	111908	GAAAGCTTTACAATAATTTCTGTGTTG GCGCAGTGTGGGACTTTAGAACAGA GGAGATAAAGATGCACACTGCGCCT	CACACTGCGCCAA CACAGAAATTATT GTAAAGCTTT
Oncostatin M	111909	GAGTAGATGTTGTTCCTGAGCCCGAG GATGTTCGGCCGGACTTTAGAACAGA GGAGATAAAGATGGGCCGAACATCC	GGCCGAACATCCT CGGGCTCAGGAA CAACATCTACT

Supplementary Table 7. Host biomarker NASBA primers

Gene	Forward primer	Reverse primer
Calprotectin *S100A9*	aattctaatacgactcactatagggagaagGGCTTTGACAGAGTGCAAGACGATGA	GTGCCCCAGCTTCACAGAGTAT
CXCL5	attctaatacgactcactatagggagaagGTCATCCAGAAAATTTTGGACGG	TCTCTGCTGAAGACTGGGAAAC
IL-8	aattctaatacgactcactatagggagaagGCCAAGGAGTGCTAAAGAACTTA	GGGTCCAGACAGAGCTCTCTT
Oncostatin M	attctaatacgactcactatagggagaagTGAACATCGAGGACTTGGAGAA	TGAGTTGTCCAGCAGCTG

Supplementary Table 8: qPCR primers and probes

Bacteria	Forward primer	Reverse primer	Probe
B. fragilis	GGCAGCGTATTAAGAGCCGTTT	GCCTGAGTTTGGTGGTAATATCTTCTG	/56-FAM/AACGCTCGC/ZEN/CCTCGTCAGGTTCAGATCGT/3IABkFQ/
B. thetaiotaomicron	CCGACTTCGGAACGCTTATAGA	TGAAACGTATGCGGTAGCTGAA	/56-FAM/AGGAGCAAC/ZEN/ACCACACAAAGCCGGTCA/3IABkFQ/
E. coli	CGGATATCTATTTCGTTTCC	GTCAGCATATTCACATACC	/56-FAM/AACCGTGAG/ZEN/CAAATCGACCGA/3IABkFQ/
B. longum	AGCTCGCTGATGGCAGT	AGCCACTGTTGTTATCGTTCAATGC	/56-FAM/ACTTGCGCG/ZEN/TCAACAGGTTCTTGTGCATCA/3IABkFQ/
B. adolescentis	ATCATGATTGCCGCGTGTTG	TTCTTTCGGCGGCTTTTGAC	/56-FAM/AGTCGTGTT/ZEN/GGCTGTGACCAGTGTGGTGA/3IABkFQ/
E. rectale	AGGCGTATTGTGGATTGTG	TTGCCCACTATAAAGCTCAAA	/56-FAM/AAGCATCTG/ZEN/CATACCTCCACGACGGT/3IABkFQ/
R. hominis	GGGCATTACCGTGGATGC	GACTTCAGCTTGTCCATATCCG	/56-FAM/CGACGGCAG/ZEN/ACTTTCCGTGGACGAGAA/3IABkFQ/
C. difficile (toxin B)	AGACTGATGAGGGATTTAG	CTCTTCAGTTATATGATTAGCA	/56-FAM/AAAGAAACT/ZEN/GGAGAATCTATATTTGTAGAAACTGA/3IABkFQ/
F. prausnitzii	GAAAACGTTGACTTGCGGCTAT	CTATTCCAAAACGCACAGCAG	/56-FAM/CTTCACAGG/ZEN/GTTTGACCGCCTATCGCAGAA/3IABkFQ/
H. sapiens Calprotectin S100A9	ATGCTGATGGCGAGGCTAA	CGAGGCCTGGCTTATGG	/56-FAM/TCCCACGAG/ZEN/AAGATGCACGAGGGTGAC/3IABkFQ/
H. sapiens CXCL5	CCGCTGCTGTGTTGAGAG	CCTATGGCGAACACTTGCAGATTAC	/56-FAM/AGCTGCGTT/ZEN/GCGTTTGTTTACAGACCACGC/3IABkFQ/
H. sapiens IL-8	AAACCACCGGAAGGAACCA	GCTGCAGAAATCAGGAAGGC	/56-FAM/AGCCACGGC/ZEN/CAGCTTGGAAGTCATGT/3IABkFQ/
H. sapiens Oncostatin M	Proprietary commercially available assay, see methods.		

Supplementary Table 9: Targeted species-specific gene regions (NASBA standard sequences)

Bacteria	Gene	Sequence
B. fragilis	DUF4834 domain-containing protein	ATGTTTCACATTTTAGGATTTTTATTCATTATTGTCATAGCC<br>GTTATAATCATCGGATTGGCCCTTGTAGGCAGCGTATTAAG<br>AGCCGTTTTCGGACTTGGAAAACGCTCGCCCTCGTCAGGT<br>TCAGATCGTAACGGACCCAATAATAATTCAGGAAGCAGAAG<br>ATATTACCACCAAACTCAGGCTAATGATAAAGAAGAAATCA<br>TCACTGGGACAGGAG
B. thetaiotaomicron	hypothetical protein SAMN02910322_01913	ATGCATGCATACATTATCCAACAACTAACAAGAATTATATTG<br>TTTATCACTATCGGTTTGCCTATAGGACTAAAAAGTTTTGCC<br>CAAGAAACAAAACGTTTCTATATGGAACTGGACACTCCCCG<br>CAATGGAGCCAAAGCAGGACAAGAGCTTGAATTAAAATACA<br>TCAGCACAGCCGATTTCGATTCTGTATCTCCACCCGACTTC<br>GGAACGCTTATAGAAACAGTTGAAGGAGCAACACCACACA<br>AAGCCGGTCATACAGTAAAAAACGGCATATTGACAGATATC<br>TACGAGCAGGGATTCAGCTACCGCATACGTTTCAAGAAGC<br>CAGGAAACACCAAACTACCTCTGGCATCCATCAAGGCAAA<br>CGGAAAGGAATACGAAACACCTCTGACCAGTGTATGGGTA<br>CATCCGGTCGATACCAATATCGACAGTGTAAAATGCAGCAT<br>TCAGCTGGAGGATTCTTATCGCAAAGGAGTTTTCACTGCCA<br>TCGGGATCTGTCTCTTAATCGCCTGGTTATTGATCCGCTTA<br>TCGTTTCAGAAACAAAAAAATAAAGAGACAGGATAA
E. coli	AraC family transcriptional regulator	TCAAACTACGACGTCATCATTTAGCCAGATGTATGAAGAAT<br>TTTAAGACGGATATCTATTTCGTTTCCACGTTTGAACCGTCA<br>ACAAAATCGGTCGATTTGCTCACGGTTGAAACTTTTGCTGG<br>TACGGTATGTGAATATGCTGACATGCCAAAAGAGTGGACA
B. longum	hypothetical protein (L,D-transpeptidase catalytic domain-containing)	CCGCAAATCAATCCCGAGCTCGCTGATGGCAGTGTGGTAG<br>ATAAGGCGCTGAACATTGATGCACAAGAACCTGTTGACGC<br>GCAAGTCGCATTGAACGATAACAACAGTGGCTTCAACGTTA<br>TTGCGGGAGAAAATGGCCAGGGAGCCAACGCCACCAGCA<br>TTGCGAAGCAAGCCATTTCCACAGTGGAGTCATTGGGCAG<br>TGTCCAGCCACAGACCGTTCGGGTGGAACTCGACGTA
B. adolescentis	hypothetical protein LU08_05010 (DUF2142 domain containing)	ATGTGCAAGTTTGTGTACATGCCATTGGTTTTGCTGGTTATT<br>CCGTTGATGTTTGATCGAGTTTCCGGTCGCTGGCGTGTGA<br>ATCGTGGTCGGGCTGTGCCATTGTTGATAGGCGTGGTTGC<br>GTCTGGTATATGGACCATATTCTGGTTGGGCGTCAATGCTT<br>GGTATACGAATTGTCCGATGCTGGTTTCGTACAAACAAATG<br>AGCGAGCGCAAGCATGCATTGTTGACGGATCCCGTTGCCA<br>TGTTGGATGCTGTGAAGAACATTGCATGGGCGATAATGCAT<br>GCTCAATCGAATATGAATAACAGGACGGACAGCATCATGAT<br>TGCCGCGTGTTGGTTGGCAATTGTGGTTTCCGTAGTCGTG<br>TTGGCTGTGACCAGTGTGGTGAATGCTTGTGTCAAAAGCC<br>GCCGAAAGAATCCGGTTAGGACTGCCAATGGTTCTATAAAT<br>GCATGCGGTGTGCTTTCCTTACCATACGCATGGTTGATCGC<br>GGTGGTCTGTATCGGAGATATCCTACTGATTTACCTTGCCT<br>TGTGACTGCAATACGATGCGGATGGGCTAATCGGAGTCGA<br>CGGTATGCAATTCAGGTATTTCCTTCCTTATGCGCCTTTGTT<br>TGCATTCGTAATGTTGGAATCGGGAAGGCGGTTGCTGAAA<br>CAGTGA

E. rectale	phosphatase PAP2 family protein	ATGGACTGGGAATTTGACATCCTATATGCAATTCAGAGCAT<br>CAGAACACCGTTTTTAGACAAGCTAATGGCGTTTTTATCCA<br>CCATCGGAAATGCAGGCGTATTGTGGATTGTGATAGGTGT<br>AGTGCTTTGTATTTCAAAAAAATACCGTCGTGGAGGTATGC<br>AGATGCTTTCGGCAGAGCTTTTGAGCTTTATAGTGGGCAAT<br>CTGATAATAAAAAATATGGTCGACAGATTGAGACCATGTCA<br>GATAGATAAGACAGTCAGTCTTATTGTAAAGATACCGTTTG<br>ACTCATCATTTCCTTCGGGACACACGTTAAACGGCATAACA<br>GCGGCAGTGACACTTATGTTTAT
R. hominis	hypothetical protein	ATTCCACGGACAGCGCCACGAAGGTCAGCCCGTCGATCTC<br>AACCGCGAAGGATGACGCGAAGACACTGACCCGGATTGAG<br>AGTGCGGCGGAGAGTACGGAGAAGTCGCTTTCCAAGCTGA<br>CAGCAACCGGAAAGGATTCTGTGTTCAACAAAGTGGAGAA<br>GACCGCGGAGGACGGCACGAAGACGCAGGAGTATGACCG<br>TGATGCCATCTATAAGGCGGTGAAGTCGTATGTGGATGATT<br>ACAATTCACTGCTGGATCGGGCGGACGATTCTAAGACGAA<br>GAGCATTCTGCGTGCGGCGAACTCTTTGAAGAGCAACGCC<br>AGAGCGAATGAGAAGACGTTGGAGAAAGCGGGCATTACCG<br>TGGATGCCGACGGCAGACTTTCCGTGGACGAGAAGAGCTT<br>TGAGAAGGCGGATATGGACAAGCTGAAGTCACTGTTTACA<br>ACGCACGGATCTTATGCGACGCAGACGAATGTGGATCTCC<br>TGAAGATCGCATC
C. difficile	toxin B (*tcdB*)	AGACTGATGAGGGATTTAGTATAAGATTTATTAATAAAGAAA<br>CTGGAGAATCTATATTTGTAGAAACTGAAAAAACAATATTCT<br>CTGAATATGCTAATCATATAACTGAAGAG
F. prausnitzii	hypothetical protein FAEPRAA2165_01415	ATGAAAAATACGGAAAAACTTGACAAAACGCTTGAGAAAAC<br>GTTGACTTGCGGCTATAATAGAAACGTGTTATTCTGCGATA<br>GGCGGTCAAACCCTGTGAAGTTTGGGCTGCTGTGCGTTTT<br>GGAATAG

Supplementary Table 10. Host biomarker mRNA standard sequences

Gene	mRNA standard sequence
Calprotectin<br>*S100A9*	CTCTGTGTGGCTCCTCGGCTTTGACAGAGTGCAAGACGATGACTTG<br>CAAAATGTCGCAGCTGGAACGCAACATAGAGACCATCATCAACACC<br>TTCCACCAATACTCTGTGAAGCTGGGGCACCCAGACACCCTGAACC<br>AGGGGGAATTCAAAGAGCTGGTGCGAAAAGATCTGCAAAATTTTCT<br>CAAGAAGGAGAATAAGAATGAAAAGGTCATAGAACACATCATGGAG<br>GACCTGGACACAAATGCAGACAAGCAGCTGAGCTTCGAGGAGTTCA<br>TCATGCTGATGGCGAGGCTAACCTGGGCCTCCCACGAGAAGATGC<br>ACGAGGGTGACGAGGGCCCTGGCCACCACCATAAGCCAGGCCTCG<br>GGGAGGGCACCCCCTAAGACCACAGTGGCCAAGATCACAGTGGCC<br>ACGGCCACGGCCACAGTCATGGTGGCCACGGCCACAGCCACTAAT<br>CAGGAGGCCAGGCCACCCTGCCTCTACCCAACCAGGGCCCCGGG
CXCL5	ATGAGCCTCCTGTCCAGCCGCGCGGCCCGTGTCCCCGGTCCTTCG<br>AGCTCCTTGTGCGCGCTGTTGGTGCTGCTGCTGCTGACGCAG<br>CCAGGGCCCATCGCCAGCGCTGGTCCTGCCGCTGCTGTGTTGAGA<br>GAGCTGCGTTGCGTTTGTTTACAGACCACGCAAGGAGTTCATCCCA<br>AAATGATCAGTAATCTGCAAGTGTTCGCCATAGGCCCACAGTGCTC<br>CAAGGTGGAAGTGGTAGCCTCCCTGAAGAACGGGAAGGAAATTTGT<br>CTTGATCCAGAAGCCCCTTTTCTAAAGAAAGTCATCCAGAAAATTTT<br>GGACGGTGGAAACAAGGAAAACTGATTAAGAGAAATGAGCACGCAT<br>GGAAAAGTTTCCCAGTCTTCAGCAGAGAAGTTTTCTGGAGGTCTCT<br>GAACCCAGGGAAGACAAGAAGGAAAGATTTTGTTGTTGTTTGTTTAT<br>TTGTTTTTCCAGTAGTTAGCTTTCTTCCTGGATTCCTCACT
IL-8	GAGGGTGCATAAGTTCTCTAGTAGGGTGATGATATAAAAAGCCACC<br>GGAGCACTCCATAAGGCACAAACTTTCAGAGACAGCAGAGCACACA<br>AGCTTCTAGGACAAGAGCCAGGAAGAAACCACCGGAAGGAACCATC<br>TCACTGTGTGTAAACATGACTTCCAAGCTGGCCGTGGCTCTCTTGG<br>CAGCCTTCCTGATTTCTGCAGCTCTGTGTGAAGGTGCAGTTTTGCCA<br>AGGAGTGCTAAAGAACTTAGATGTCAGTGCATAAAGACATACTCCAA<br>ACCTTTCCACCCCAAATTTATCAAAGAACTGAGAGTGATTGAGAGTG<br>GACCACACTGCGCCAACACAGAAATTATTGTAAAGCTTTCTGATGGA<br>AGAGAGCTCTGTCTGGACCCCAAGGAAAACTGGGTGCAGAGGGTT<br>GTGGAGAAGTTTTTGAAGAGGGCTGAGAATTCATAAAAAAATTCATT<br>CTCTGTGGTATCCAAGAATCAGTGAAGATGCCAGTGAAACTTCAAG<br>CAAATCTACTTCAACACTTCATGTATTGTGTGGGTCTGTTGTAGGGT<br>TGCCAGATGCAATACAAGATTCCTGGTTAAATTTGAATTTCAGTAAA<br>CAATGAATAGTTTTTCATTGTACCAGGATCC
Oncostatin M	CTGAGGGGGCTGGGCAGGCGGGGCTTCCTGCAGACCCTCAATGCC<br>ACACTGGGCTGCGTCCTGCACAGACTGGCCGACTTAGAGCAGCGC<br>CTCCCCAAGGCCCAGGATTTGGAGAGGTCTGGGCTGAACATCGAG<br>GACTTGGAGAAGCTGCAGATGGCGAGGCCGAACATCCTCGGGCTC<br>AGGAACAACATCTACTGCATGGCCCAGCTGCTGGACAACTCAGACA<br>CGGCTGAGCCCACGAAGGCTGGCCGGGGGGCCTCTCAGCCGCCC<br>ACCCCCACCCCTGCCTCGGATGCTTTTCAGCGCAAGCTGGAGGGC<br>TGCAGGTTCCTGCATGGCTACCATCGCTTCATGCACTCAGTGGGGC<br>GGGTCTTCAGCAAGTGGGGGGAGAGCCCGAACCGGAGCCGGAGA<br>CACAGCCCCCACCAGGCCCTGAGGAAGGGGGTGCGCAGGACCAG<br>ACCCTCCAGGAAAGGCAAGAGACTCATGACCAGGGGACAGCTGCC<br>CCGGTA

Supplementary Table 11: qPCR performance characteristics

Bacteria	Tm (ºC)	Efficiency
B. fragilis	60	0.9742-0.9983
B. thetaiotaomicron	60	0.9215-1.0031
E. coli	55	0.933-0.9968
B. longum	60	0.9108-0.9445
B. adolescentis	60	0.9815-0.994
E. rectale	60	0.9526-0.9587
R. hominis	60	0.991-1.0198
C. difficile (toxin B)	48	0.9153-1.0229
F. prausnitzii	60	0.9492-1.0216
H. sapiens Calprotectin S100A9	60	1.0278
H. sapiens CXCL5	60	1.0297
H. sapiens IL-8	60	1.0665
H. sapiens Oncostatin M	60	1.0139

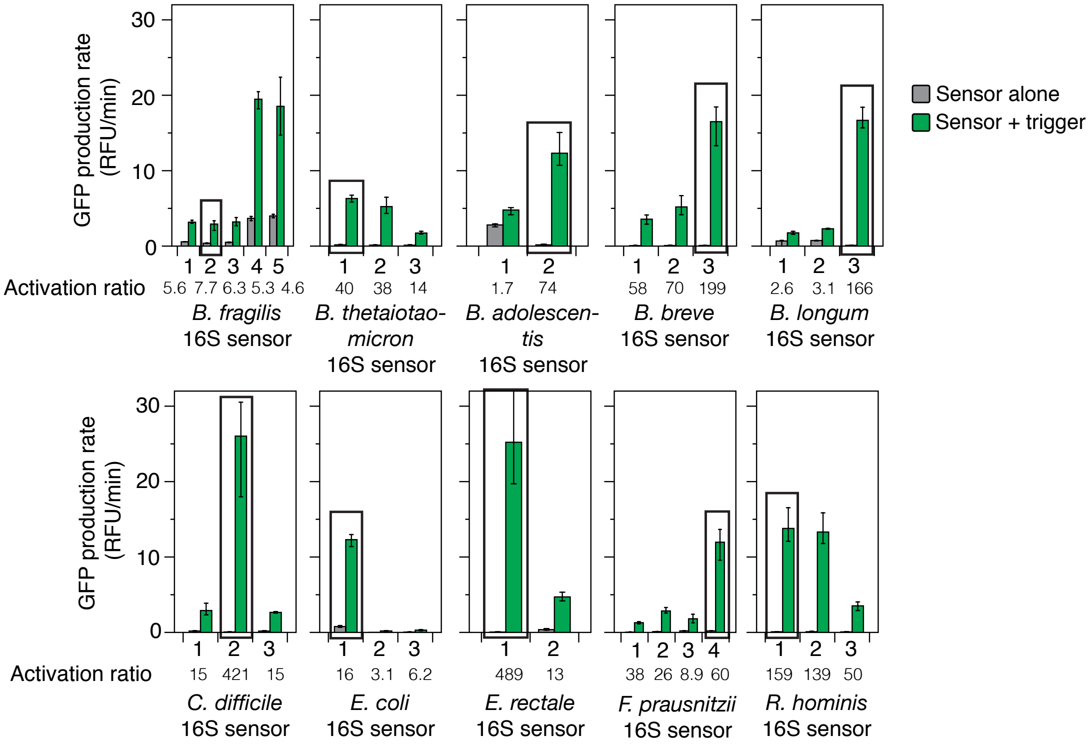

Supplementary Figure 1. 16S toehold switch sensor screen. Candidate toehold switch sensors were tested in paper-based reactions with and without 2 μM trigger RNA (36 nucleotides). Data represent mean GFP production rates from three technical replicates. Error bars represent high and low values of the three replicates. The activation ratio for each sensor candidate was calculated by dividing the mean 'sensor + trigger' GFP production rate by the mean 'sensor alone' GFP production rate. Sensors were chosen for highest activation ratio and lowest 'sensor alone' GFP production rate. Boxes indicated selected sensors shown in Figure 2b.

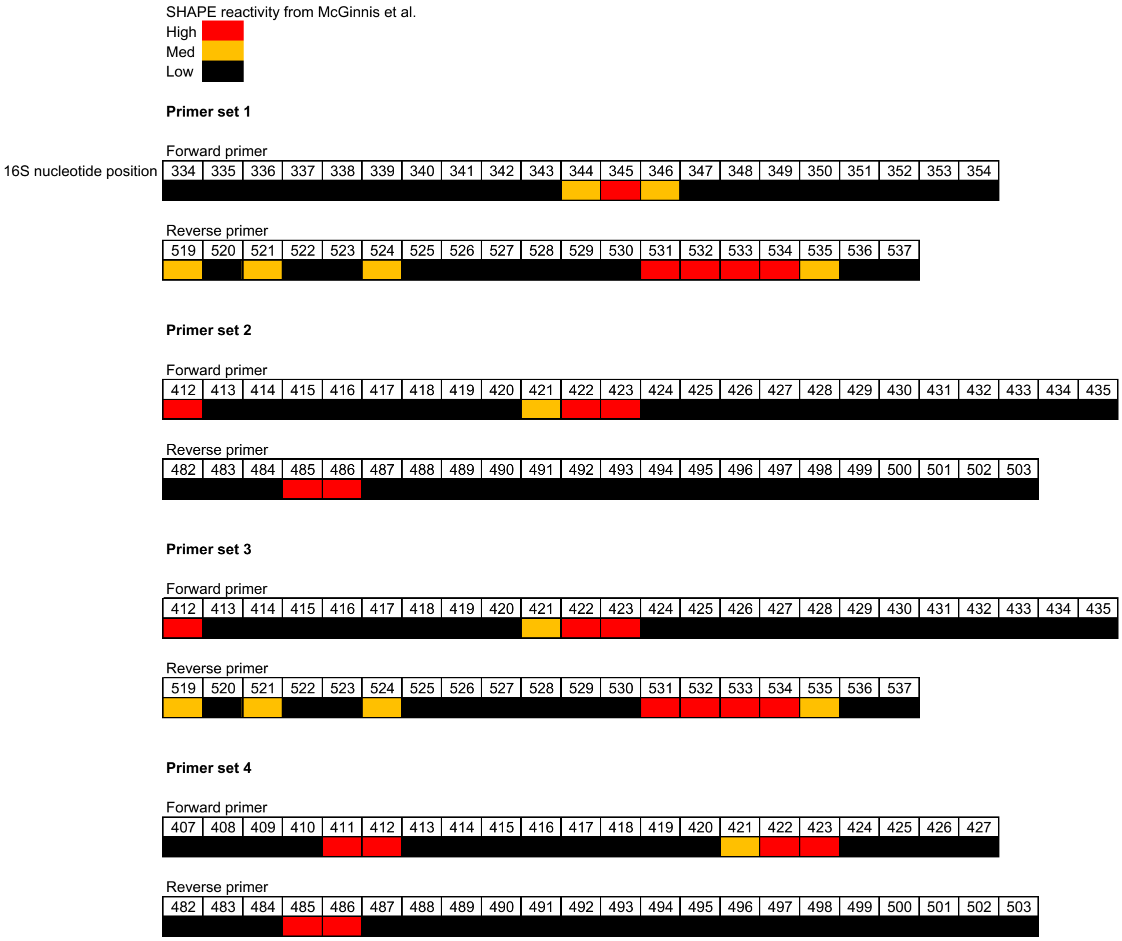

Supplementary Figure 2. Chemical structure probing data for *E. coli* 16S NASBA primers. NASBA primers were mapped to SHAPE reactivities of *E. coli* 30S subunits from McGinnis et al[1]. Reactivities indicate the structural accessibility of the individual nucleotides. Increased accessibility for the forward NASBA primer increased amplification efficiency.

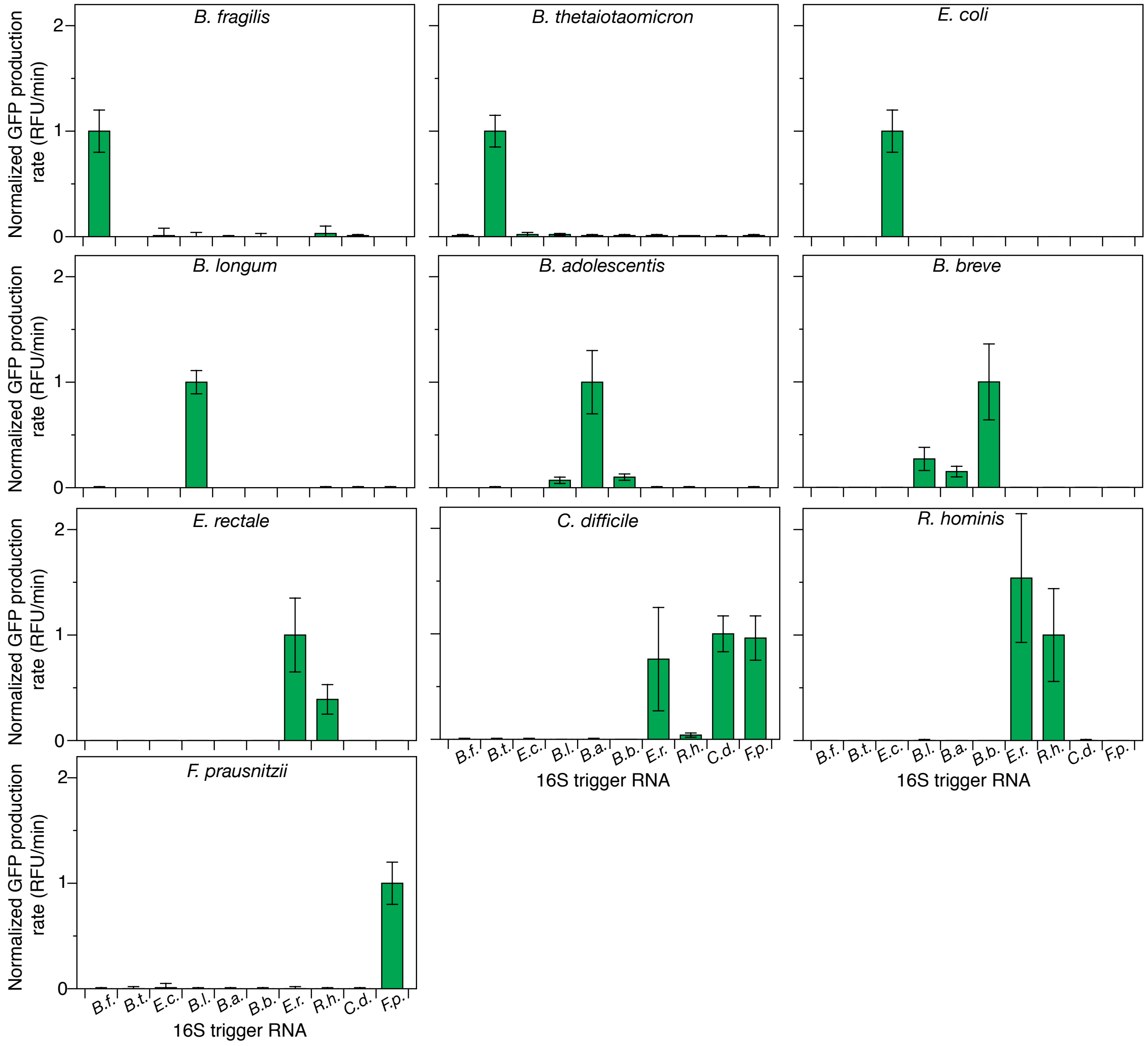

Supplementary Figure 3. 16S sensor orthogonality data. These data are represented in Figure 2e. Each sensor was challenged with 2 μM of trigger RNAs from each species representing what would be amplified in a NASBA reaction. GFP production rates for an individual sensor were normalized to the production rate of the sensor plus its cognate trigger. Data represent mean ± s.d. from six replicates (two biological replicates x three technical replicates).

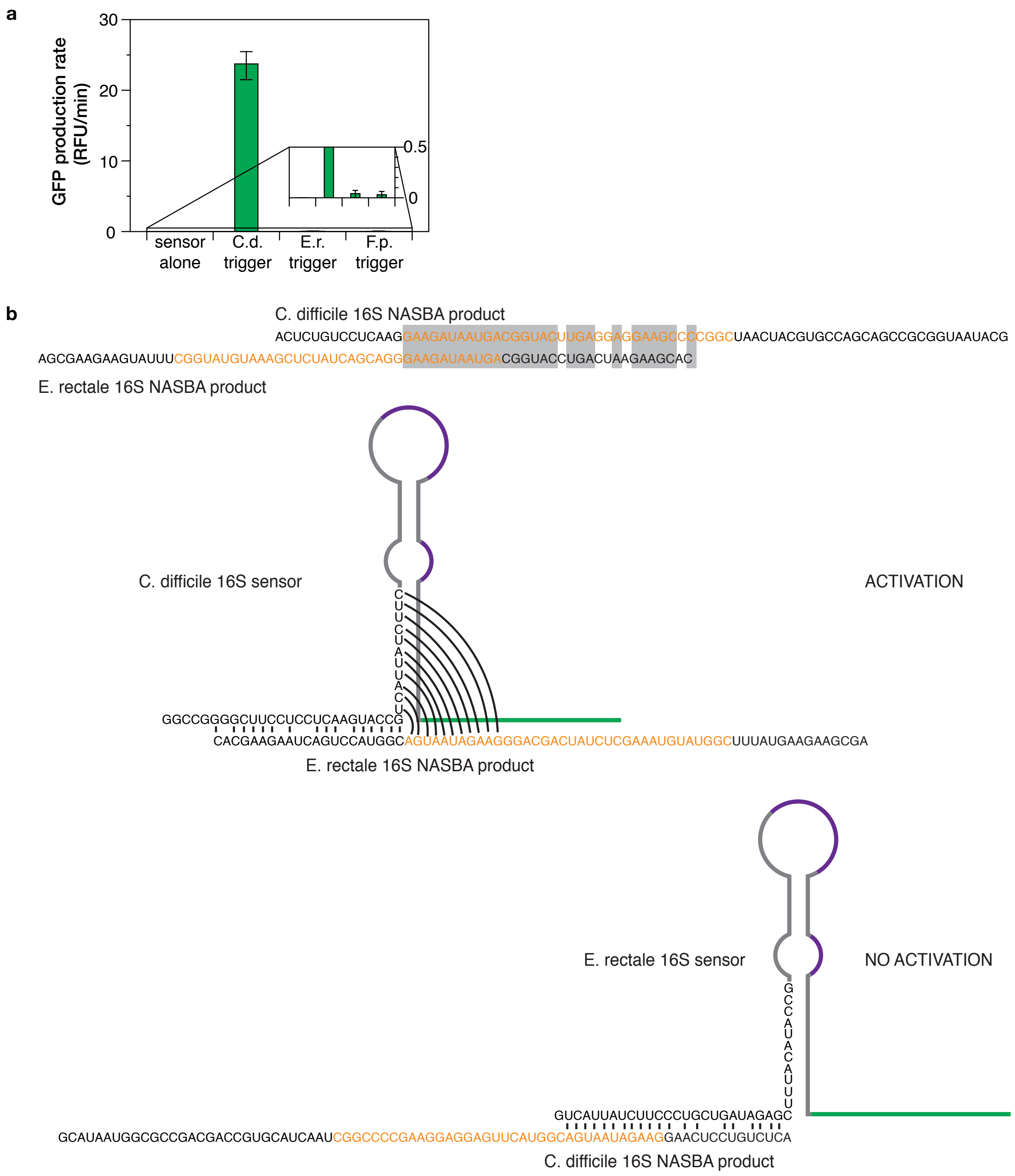
a
GFP production rate (RFU/min)
30
20
10
0
0.5
0
sensor alone
C.d. trigger
E.r. trigger
F.p. trigger
b
C. difficile 16S NASBA product
ACUCUGUCCUCAAGGAAGAUAAUGACGGUACUUGAGGAGGAAGCCCCGGCUAACUACGUGCCAGCAGCCGCGGUAAUACG
AGCGAAGAAGUAUUUCGGUAUGUAAAGCUCUAUCAGCAGGGAAGAUAAUGACGGUACCUGACUAAGAAGCAC
E. rectale 16S NASBA product
C. difficile 16S sensor
ACTIVATION
CUUCUAUUACU
GGCCGGGGCUUCCUCCUCAAGUACCG
CACGAAGAAUCAGUCCAUGGCAGUAAUAGAAGGGACGACUAUCUCGAAAUGUAUGGCUUUAUGAAGAAGCGA
E. rectale 16S NASBA product
E. rectale 16S sensor
NO ACTIVATION
GCCAUACAUUU
GUCAUUAUCUUCCCUGCUGAUAGAGC
GCAUAAUGGCGCCGACGACCGUGCAUCAAUCGGCCCCGAAGGAGGAGUUCAUGGCAGUAAUAGAAGGAACUCCUGUCUCA
C. difficile 16S NASBA product

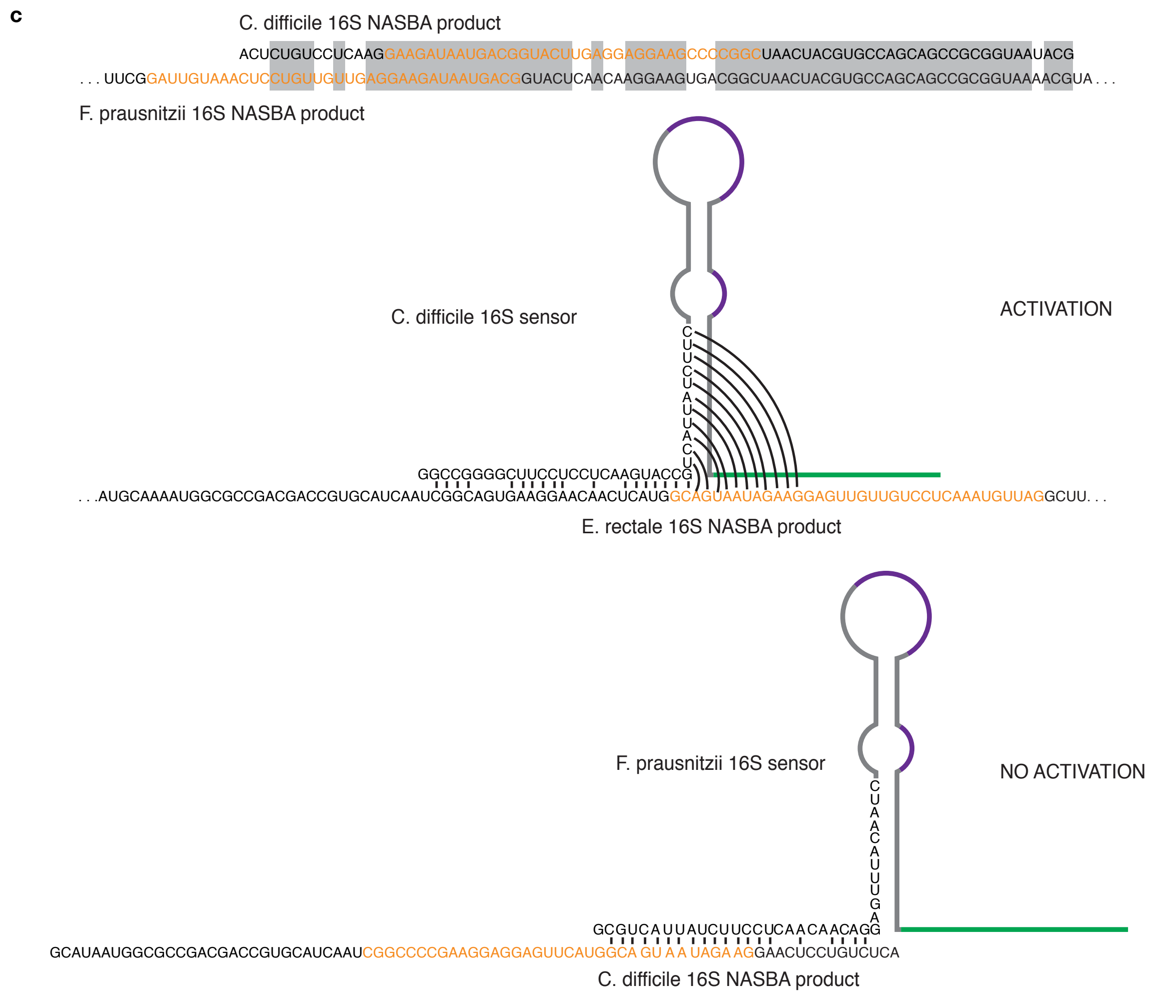

Supplementary Figure 4. *C. difficile* 16S sensor alignment. (a) *C. difficile* 16S sensor tested against the *E. rectale* and *F. prausnitzii* 36 nucleotide trigger RNAs (2 µM). Data represent mean values from three technical replicates. Error bars represent high and low values of the three replicates. (b) Alignment of the *C. difficile* and *E. rectale* 16S NASBA product. Diagrams indicate theoretical activation of the *C. difficile* sensor by the *E. rectale* product, but no activation of the *E. rectale* sensor by the *C. difficile* product. (c) Alignment of the *C. difficile* and *F. prausnitzii* 16S NASBA product. Diagrams indicate theoretical activation of the *C. difficile* sensor by the *F. prausnitzii* product, but no activation of the *F. prausnitzii* sensor by the *C. difficile* product.

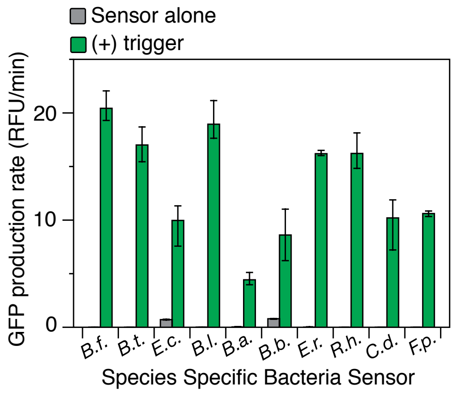

Supplementary Figure 5. Species-specific toehold switch sensor function. Best performing species-specific sensors. Sensors were tested in paper-based reactions with and without 2 μM trigger RNA (36 nucleotides). Data represent mean values from three technical replicates. Error bars represent high and low values of the three replicates.

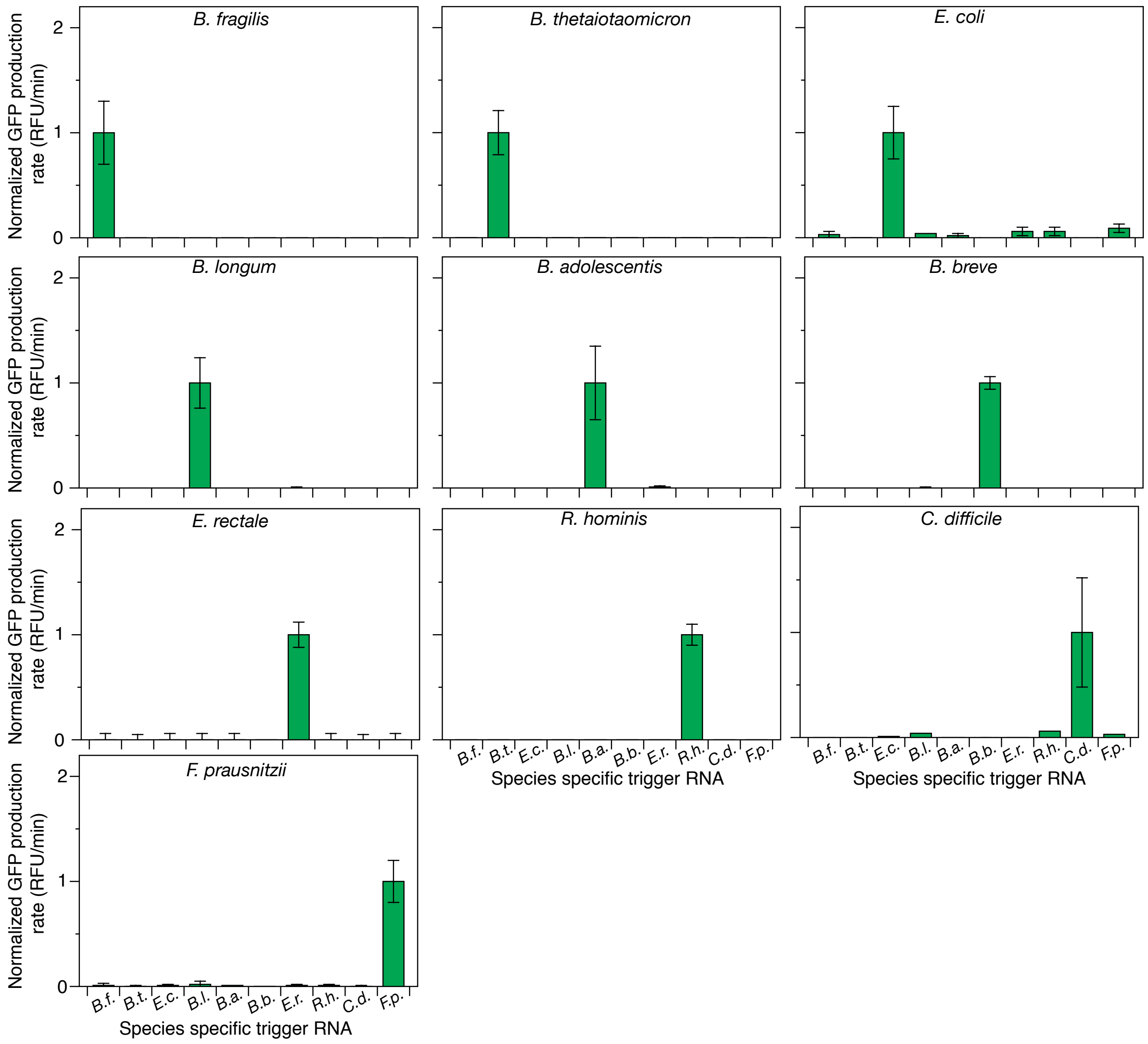

Supplementary Figure 6. Species-specific sensor orthogonality. These data are represented in Figure 3c. Each sensor was challenged with 2 μM of trigger RNAs from each species representing what would be amplified in a NASBA reaction. GFP production rates for an individual sensor were normalized to the production rate of the sensor plus its cognate trigger. Data represent mean ± s.d. from six replicates (two biological replicates x three technical replicates).

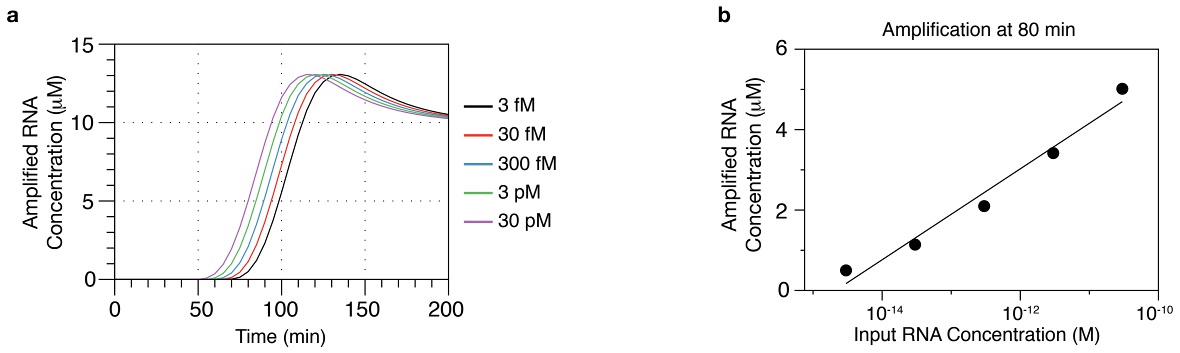

Supplementary Figure 7. Mathematical model of NASBA process. Differential equations describing the chemical reactions in NASBA were solved in Matlab (see Supplementary Note). (a) NASBA reaction time courses for varying input mRNA concentrations. The model suggests that mRNA standards ranging between 3 fM and 30 pM could be amplified to μM RNA concentrations, which is within the detection limit of toehold switch sensors. (b) Input RNA concentration vs. amplified RNA concentration from (a) at the 80-minute time point. The model predicts that if NASBA reactions are stopped before completion, amplified RNA concentrations can be distinguished from one another and quantified using a log-linear fit.

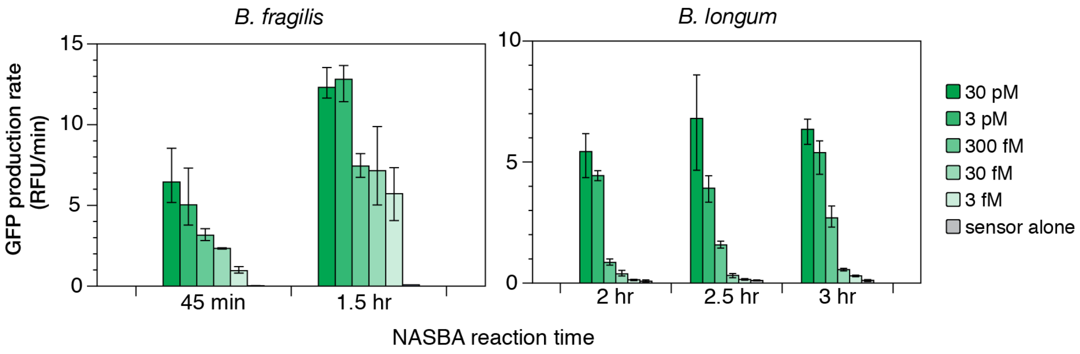

Supplementary Figure 8. NASBA time courses. NASBA reactions of varying times were performed on mRNA standards and tested using species-specific mRNA sensors for *B. fragilis* and *B. longum*. Data represent mean values from three technical replicates. Error bars represent high and low values from the three replicates.

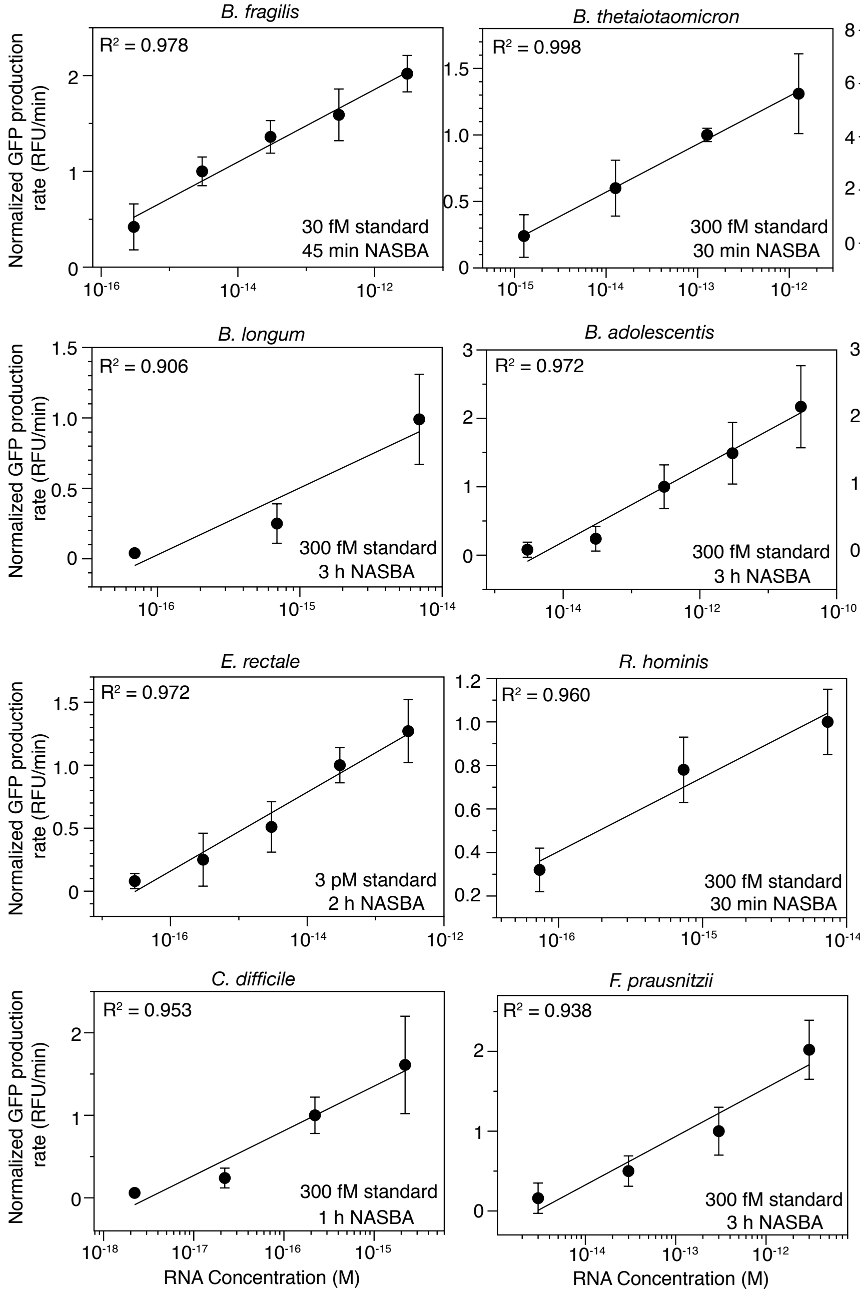

	A	B
B.f.	0.165	6.403
B.t.	0.157	5.624
E.c.	0.819	28.898
B.l.	0.206	8.578
B.a.	0.236	7.607
B.b.	0.322	9.791
E.r.	0.136	5.063
R.h.	0.148	5.845
C.d.	0.235	9.465
F.p.	0.235	7.773

Supplementary Figure 9. Species-specific bacterial calibration curves. Species-specific mRNA calibration curves were determined by performing three separate runs of mRNA standards ranging from 3 fM to 30 pM as in Figure 4a. GFP production rates from individual runs were normalized to a single standard. Normalized values for each standard concentration were then averaged across runs and fit to the equation Normalized GFP production = A*ln(concentration) + B. The x-axis concentrations were then corrected for differences between running standards in yeast tRNA or total stool RNA backgrounds by using standards in stool RNA to calculate the apparent concentration of the normalization standard in yeast tRNA. The NASBA reaction time and normalization standard concentration for each species is indicated in each plot. Data represent mean ± s.d. from 27 replicates (nine biological replicates (NASBA reactions) x three technical replicates (paper-based reactions)).

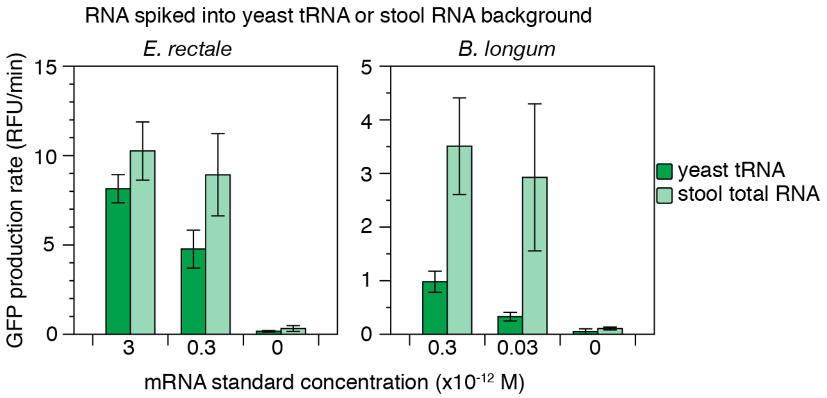

Supplementary Figure 10. mRNA detection in stool RNA background. A commercial stool sample was processed for total RNA using the RNeasy PowerMicrobiome kit and diluted to 50 ng/μl. Species-specific mRNA standards were spiked into both yeast tRNA and stool total RNA, and tested in NASBA and paper-based reactions. Data represent mean ± s.d. from nine replicates (three biological replicates (NASBA reactions) x three technical replicates (paper-based reactions)).

Clinical sample	Bacteria	Paper-based copies per 50 ng stool RNA	Paper-based error	RT-qpcr copies per 50 ng stool RNA	RT-qpcr error
S1	B.f.	610.3	173.4	11.3	1.7
S1	B.t.	0.0	0.0	0.7	0.1
S1	E.c.	0.0	0.0	0.0	0.0
S1	B.l.	0.0	0.0	0.0	0.0
S1	B.a.	0.0	0.0	0.0	0.0
S1	R.h.	0.0	0.0	0.0	0.0
S1	F.p.	0.0	0.0	1.1	9.6
S2	B.f.	0.0	0.0	0.0	0.0
S2	B.t.	0.0	0.0	0.0	0.0
S2	E.c.	0.0	0.0	0.0	0.0
S2	B.l.	26452.6	5195.7	201.7	22.3
S2	B.a.	0.0	0.0	0.0	0.0
S2	R.h.	0.0	0.0	0.0	0.0
S2	F.p.	0.0	0.0	0.0	0.0
S3	B.f.	0.0	0.0	0.0	0.0
S3	B.t.	0.0	0.0	0.0	0.0
S3	E.c.	0.0	0.0	0.0	0.0
S3	B.l.	0.0	0.0	0.0	0.0
S3	B.a.	0.0	0.0	0.0	0.0
S3	E.c.	955.4	207.1	128.7	2.2
S3	R.h.	97867.3	35577.7	2885.0	172.8
S3	F.p.	45803.2	14607.6	2655.0	247.0
S4	B.f.	0.0	0.0	2.8	1.9
S4	B.t.	0.0	0.0	0.0	0.0
S4	E.c.	0.0	0.0	0.0	0.0
S4	B.l.	0.0	0.0	0.0	0.0
S4	B.a.	0.0	0.0	0.0	0.0
S4	E.r	0.0	0.0	2.3	0.8
S4	R.h.	62992.0	15645.2	3846.7	133.3
S4	F.p.	0.0	0.0	61.3	13.5
S5	B.f.	0.0	0.0	0.0	0.0
S5	B.t.	0.0	0.0	0.0	0.0
S5	E.c.	0.0	0.0	0.0	0.0
S5	B.a.	0.0	0.0	0.0	0.0
S5	E.r.	46.0	0.9	8.2	1.9
S5	R.h.	0.0	0.0	0.0	0.0
S5	F.p.	0.0	0.0	0.6	7.4
S6	B.f.	0.0	0.0	0.0	0.0
S6	B.t.	0.0	0.0	0.0	0.0
S6	E.c.	0.0	0.0	0.0	0.0
S6	B.l.	0.0	0.0	0.0	0.0
S6	B.a.	6451.0	361.2	40.6	4.9
S6	R.h.	0.0	0.0	0.0	0.0
S6	F.p.	0.0	0.0	0.0	0.0

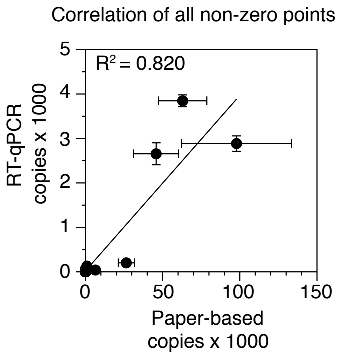

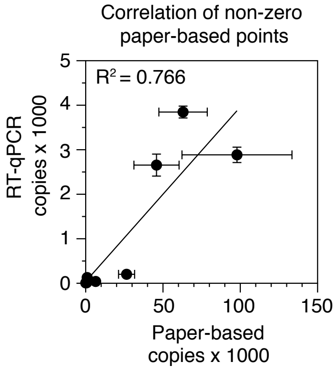

Supplementary Figure 11. Quantification of species-specific mRNAs in clinical samples. These data are represented in Figure 4d-e. Table indicates mean values and s.d. of data included in the color-map in Figure 4d. Highlighted values were used in the upper-correlation plot. Only non-zero values as determined by the paper-based system were used in the lower-correlation plot. Paper-based error bars represent s.d. from nine replicates (three biological replicates (NASBA reactions) x three technical replicates (paper-based reactions)). RT-qPCR error bars represent s.d. from six replicates (two biological replicates (RT reactions) x three technical replicates (qPCR reactions)).

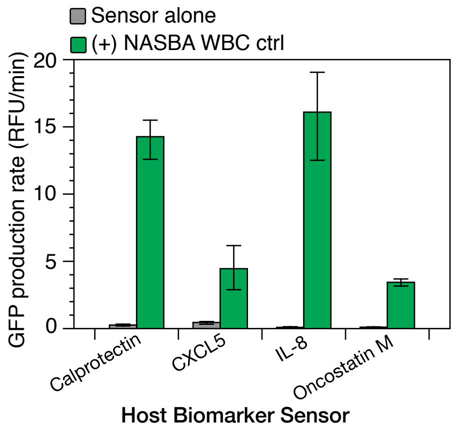

Supplementary Figure 12. Host biomarker sensor and NASBA primer validation. Host biomarker sensors and NASBA primers were tested on total RNA extracted from human white blood cells (WBC ctrl, Takara Bio 636592). Outputs from NASBA reactions were used to activate toehold switch sensors in paper-based reactions. Data represent mean values of three technical replicates. Error bars represent high and low values of the three replicates.

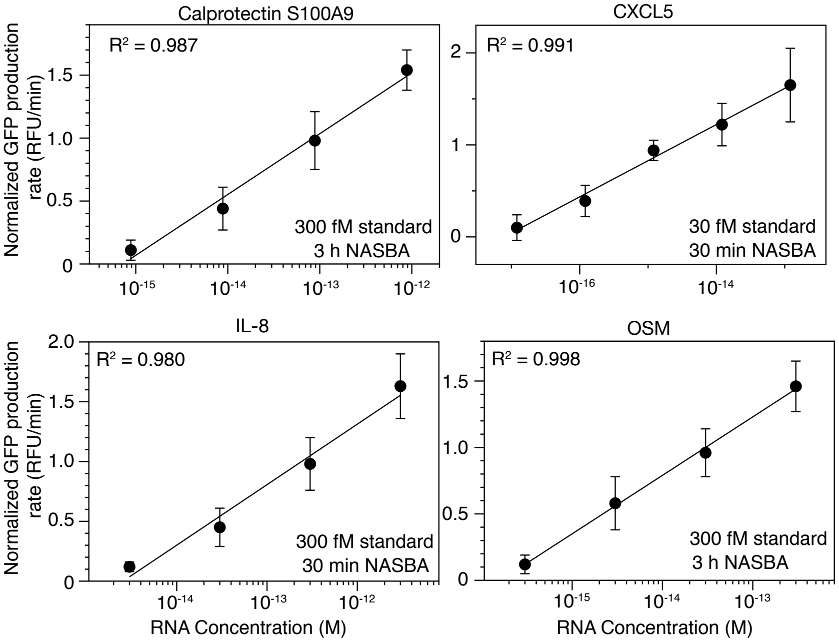

GFP production = A ln(conc) + B

	A	B
S100A9	0.210	7.314
CXCL5	0.171	6.723
IL-8	0.220	7.385
OSM	0.191	6.950

Supplementary Figure 13. Host biomarker calibration curves. Host biomarker mRNA calibration curves were determined by performing three separate runs of mRNA standards ranging from 3 fM to 30 pM. GFP production rates from individual runs were normalized to a single standard. Normalized values for each standard concentration were then averaged across runs and fit to the equation Normalized GFP production = A*ln(concentration) + B. The x-axis concentrations were then corrected for differences between running standards in yeast tRNA or total stool RNA backgrounds by using standards in stool RNA to calculate the apparent concentration of the normalization standard in yeast tRNA. The NASBA reaction time and normalization standard concentration for each species is indicated in each plot. Data represent ± s.d. from 27 replicates (nine biological replicates (NASBA reactions) x three technical replicates (paper-based reactions)).

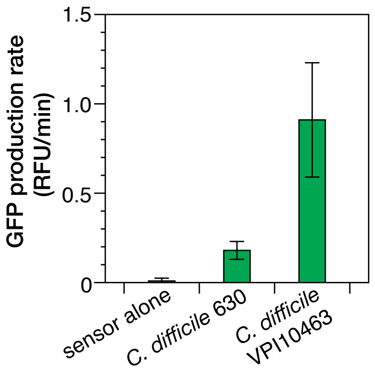

Supplementary Figure 14. *C. difficile* toxin sensor and NASBA validation. Toxin B sensor and NASBA primers were tested on total RNA extracted from cultures of *C. difficile* 630 and *C. difficile* VPI 10463. NASBA reactions were performed on 25 ng of total RNA for 3 hr. Outputs from NASBA reactions were used to activate toehold switch sensors in paper-based reactions. Data represent mean ± s.d. from nine replicates (three biological replicates (NASBA reactions) x three technical replicates (paper-based reactions)).

Supplementary Table 12. *C. difficile* toxin DNA qPCR results. Samples from Figure 6c were tested for toxin B DNA using the qPCR primers in Supplementary Table 8. ND = not determined. Any sample with Cq < 40 was considered positive.

Sample	Cq mean	Cq error
stool zero	ND	ND
630, M1	30.53	0.1
630, M2	30.71	0.15
VPI, M1	28.75	0.05
VPI, M2	30.78	0.04

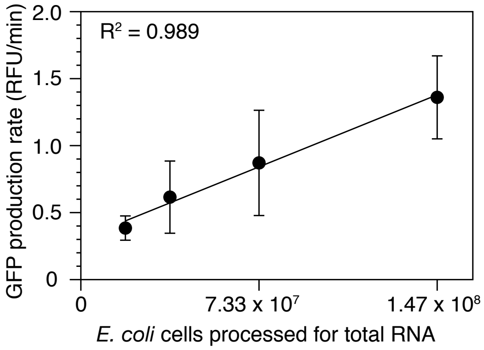

Supplementary Figure 15. Correlation of *E. coli* species-specific mRNA to cell count. Varying amounts of *E. coli* cells (1.83 x 10^7 – 1.47 x 10^8) were processed for total RNA and tested in NASBA and paper-based reactions. Processed total RNA was diluted 1:50 in 50 ng/μl yeast tRNA prior to NASBA reactions. Data represent mean ± s.d. from nine replicates (three biological replicates (NASBA reactions) x three technical replicates (paper-based reactions)).

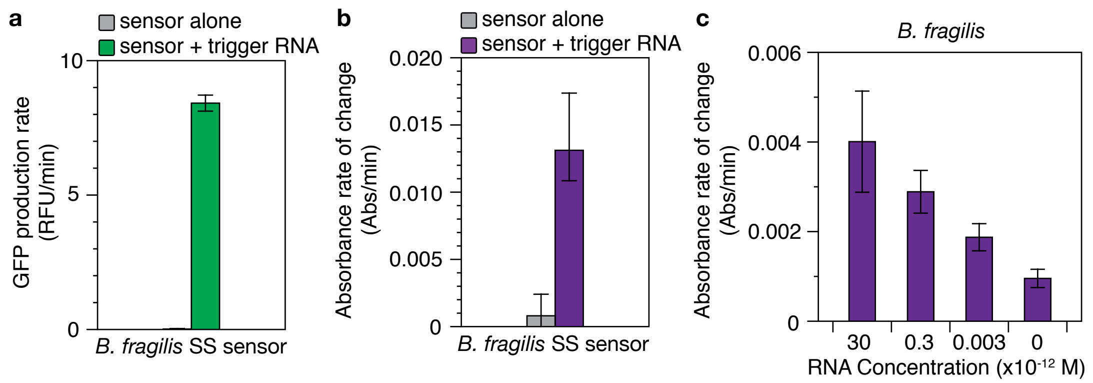

Supplementary Figure 16. Demonstration of platform using an in-house cell-free system. In-house cell-free extract and buffer were prepared (see Methods) and used to test toehold switch sensors. (a) The *B. fragilis* species-specific sensor was tested in paper-based reactions with and without 2 μM trigger RNA (36 nucleotides). Whatman GF/F glass fiber substrate (Whatman 1825047) was used instead of the standard paper substrate (Whatman, 1442-042) due to high background autofluorescence of the cell-free extract on the standard paper. (b) As a demonstration of the flexibility of the platform, toehold switch sensors were used to regulate the translation of the LacZ enzyme. The *B. fragilis* species-specific sensor was tested in paper-based reactions with and without 2 μM trigger RNA (36 nucleotides). (c) NASBA reactions were performed on *B. fragilis* mRNA standards for 45 min and tested in paper-based reactions. In (a) and (b) data represent mean of three technical replicates and error bars represent the high and low values of the three replicates. In (c) data represent mean of nine replicates (three biological replicates (NASBA reactions) x three technical replicates (paper-based reactions)) and error bars represent s.d.

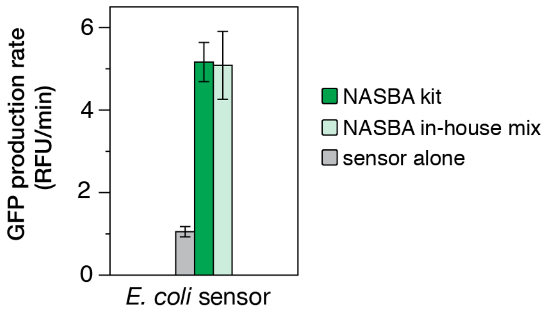

Supplementary Figure 17. Performance of NASBA reactions using individually mixed components. NASBA reactions were run on the 30 pM *E. coli* species-specific mRNA standard for 90 minutes and tested in paper-based reactions. Data represent mean ± s.d. of six replicates (two biological replicates (NASBA reactions) x three technical replicates (paper-based reactions)).

Supplementary Note 1. Toehold switch sensor design script.
The sequence for “source MRNA” was replaced with the V3 hypervariable region of 16S ribosomal RNA sequences from each species, species-specific mRNA sequences determined by our computational pipeline, or human mRNA sequences.

```
# Template for toehold design for mRNA trigger
material = rna
temperature[C] = 37    # optional units: C (default) or K
trials = 10
sodium[M] = 1.0    # optional units: M (default), mM, uM, nM, pM
dangles = some
allowmismatch = true

#target structure
structure switch = ............................(((((((((((...(((((............)))))...)))))))))))......................
structure trigger = ....................................
structure activated =
((((((((((((((((((((((((((((((((((((+...)))))))))))))))))))))))))))))))))))...(((((............))))).................................... 
#sequence domains
#series B conserved sequence GGACUUUAGAACAGAGGAGAUAAAGAUG
#Green et al linker AACCUGGCGGCAGCGCAAAAG
domain a = N11
domain b = N25
domain g = GGG
domain s = GGACUUUAGAACAGAGGAGAUAAAGAUG
domain l = N1 AACCUGGCGGCAGCGCAAAAG
#source sequence
source MRNA =
TATGGTTGTAAAGCACTTTAAGCGAGGAGGAGGCTACTTTAGTTAATACCTAGAGATAGTGG
ACGTTACTCGC
#windows from sources
window rrna_window = a b
rrna_window.source = RRNA
switch.seq = g b* a* s a l
trigger.seq = a b
activated.seq = a b g b* a* s a l
#stop condition for ensemble defect
switch.stop = 10.0
prevent = AAAA, CCCC, GGGG, UUUU
```

Supplementary Note 2. Description of NASBA Mathematical Model.

To model NASBA reactions, we assumed each reaction followed standard mass-action Michaelis-Menten kinetics. NASBA enzymes bind their cognate ligand with an affinity K_D to form a complex and produce a product at a rate of k_{cat}. The concentration of each species in the system was calculated for each time step by solving a series of differential equations that describes the change in concentration of each species over time. The differential equations describing enzyme kinetics are nonlinear by nature and thus were solved numerically. The differential equations were solved in Matlab using a stiff ODE solver with an error tolerance of 1e^-13.

Model species: Initial concentrations reflect concentrations used in experimental reactions.

**Species Name**	**Symbol**	**Initial Concentration**
mRNA	*mRNA*	1 pM
Primer 1	*P1*	250 nM
mRNA-Primer1	*mRNA-P1*	0
Reverse Transcriptase	*RT*	1 nM
mRNA-Primer1-RT	*mRNA-P1-RT*	0
Complexed ssDNA/RNA	*C*	0
RNAse H	*H*	0.05 nM
Complex-RNAse H	*C-H*	0
Single-stranded DNA	*ssDNA*	0
Primer 2	*P2*	250 nM
ssDNA–Primer 2	*ssDNA-P2*	0
T7 RNAP	*T7*	5 nM
ssDNA-Primer 2- RT	*ssDNA-P2-RT*	0
Double-stranded DNA	*dsDNA*	0
dsDNA-T7 RNAP	*dsDNA-T7*	0
dNTP	*dNTP*	20000 nM
NTP	*NTP*	10000 nM
Background RNA	*bkRNA*	700 nM
Background-Primer1	*bkRNA-P1*	0
Background-Primer 2	*bkRNA-P2*	0

Reaction rates: Rate constants were obtained from Bionumbers (http://www.bionumbers.hms.harvard.edu/)

**Name**	**Symbol**	**Value**
Primer Binding	k_p	10^6
Primer Unbinding	$k^{-1}{}_p$	10^{-4}
RT Binding	k_{rt}	10^6
RT Unbinding	$k^{-1}{}_{rt}$	10^{-4}
Synthesis	k_{syn}	0.0005 nM s^{-1} (based on turnover)
RNAse H Binding	k_h	10^5
RNAse H Unbinding	$k^{-1}{}_h$	10^{-4}

T7 Binding	k_{t7}	10^5
T7 Unbinding	k^{-1}_{t7}	10^{-4}
Degradation	k_{deg}	0.05 nM s^{-1} (based on turnover)
Background Binding	k_{pb}	$5*10^5$
Background Unbinding	k^{-1}_{pb}	10^{-4}

Chemical Reactions

Supplementary Equation 1

$$mRNA + P1 \underset{k_p}{\overset{k_p^{-1}}{\rightleftharpoons}} mRNA - P1$$

Supplementary Equation 2

$$mRNA - P1 + RT \underset{k_{rt}}{\overset{k_{rt}^{-1}}{\rightleftharpoons}} mRNA - P1 - RT + dNTP \overset{k_{syn}}{\rightarrow} C + RT + P1$$

Supplementary Equation 3

$$C + H \underset{k_h}{\overset{k_h^{-1}}{\rightleftharpoons}} C - H \overset{k_{deg}}{\rightarrow} H + ssDNA$$

Supplementary Equation 4

$$ssDNA + P2 \underset{k_P}{\overset{k_P^{-1}}{\rightleftharpoons}} ssDNA - P2$$

Supplementary Equation 5

$$ssDNA - P2 + RT \underset{k_{rt}}{\overset{k_{rt}^{-1}}{\rightleftharpoons}} ssDNA - P2 - RT + dNTP \overset{k_{syn}}{\rightarrow} dsDNA + RT + P2$$

Supplementary Equation 6

$$dsDNA + T7 \underset{k_{t7}}{\overset{k_{t7}^{-1}}{\rightleftharpoons}} dsDNA - T7 + NTP \overset{k_{syn}}{\rightarrow} mRNA$$

Differential Equations

Supplementary Equation 7: Free mRNA

$$\frac{d[mRNA]}{dt} = k_p^{-1}[mRNA - P1] + k_{syn}[T7][dsDNA][NTP] - k_p[mRNA][P1]$$

Supplementary Equation 8: Free Primer 1

$$\frac{d[P1]}{dt} = k_p^{-1}[mRNA - P1] + k_{syn}[dNTP][mRNA - P1 - RT] - k_p[mRNA][P1] - k_{pb}[bkRNA][P1] + k_{pb}^{-1}[bkRNA - P1]$$

Supplementary Equation 9: Complexed mRNA-Primer1

$$\frac{d[mRNA-P1]}{dt} = k_p[mRNA][P1] - k_p^{-1}[mRNA-P1] - k_{rt}[mRNA-P1][RT] + k_{rt}^{-1}[mRNA-P1-RT]$$

Supplementary Equation 10: Free Reverse Transcriptase

$$\frac{d[RT]}{dt} = k_{rt}^{-1}[mRNA-P1-RT] + k_{syn}[dNTP][mRNA-P1-RT] - k_{rt}[mRNA-P1][RT] - k_{rt}[ssDNA-P1][RT] + k_{rt}^{-1}[ssDNA-P2-RT] + k_{syn}[dNTP][ssDNA-P2-RT]$$

Supplementary Equation 11: Complexed mRNA-P1-RT

$$\frac{d[mRNA-P1-RT]}{dt} = k_{rt}[mRNA-P1][RT] - k_{rt}^{-1}[mRNA-P1-RT] - k_{syn}[dNTP][mRNA-P1-RT]$$

Supplementary Equation 12: mRNA-ssDNA Complex

$$\frac{d[C]}{dt} = k_{syn}[dNTP][mRNA-P1-RT] + k_h^{-1}[C-H] - k_h[H][C]$$

Supplementary Equation 13: RNAse H

$$\frac{d[H]}{dt} = k_h^{-1}[C-H] + k_{deg}[C-H] - k_h[H][C]$$

Supplementary Equation 14: Complexed mRNA-ssDNA-RNAse H

$$\frac{d[C-H]}{dt} = k_h[C][H] - k_h^{-1}[C-H] - k_{deg}[C-H]$$

Supplementary Equation 15: ssDNA

$$\frac{d[ssDNA]}{dt} = k_p^{-1}[ssDNA-P2] - k_p[ssDNA][P2] + k_{deg}[C-H]$$

Supplementary Equation 16: Free Primer 2

$$\frac{d[ssDNA]}{dt} = k_p^{-1}[ssDNA-P2] - k_p[ssDNA][P2] + k_{pb}^{-1}[bkRNA-P2] - k_{pb}[bkRNA][P2] + k_{syn}[ssDNA-P2-RT][dNTP]$$

Supplementary Equation 17: Complexed ssDNA-P2

$$\frac{d[ssDNA-P2]}{dt} = k_p[ssDNA][P2] - k_p^{-1}[ssDNA-P2]k_{rt}[ssDNA-P2][RT] + k_{rt}^{-1}[ssDNA-P2-RT]$$

Supplementary Equation 18: Free T7 RNAP

$$\frac{d[T7]}{dt} = k_{t7}^{-1}[dsDNA-T7] - k_{t7}[dsDNA][T7]$$

Supplementary Equation 19: Complexed ssDNA-P2-RT

$$\frac{d[ssDNA-P2-RT]}{dt} = k_{rt}[ssDNA-P2][RT] - k_{rt}^{-1}[ssDNA-P2-RT] - k_{syn}[ssDNA-P2-RT][dNTP]$$

Supplementary Equation 20: Free dsDNA

$$\frac{d[dsDNA]}{dt} = k_{syn}[ssDNA-P2-RT][dNTP] + k_{t7}^{-1}[dsDNA-T7] - k_{t7}[dsDNA][T7]$$

Supplementary Equation 21: Complexed dsDNA-T7

$$\frac{d[dsDNA-T7]}{dt} = k_{t7}[dsDNA][T7] - k_{t7}^{-1}[dsDNA-T7]$$

Supplementary Equation 22: dNTP

$$\frac{d[dNTP]}{dt} = -k_{syn}[dNTP][mRNA-P1-RT] - k_{syn}[dNTP][ssDNA-P2-RT]$$

Supplementary Equation 23: NTP

$$\frac{d[NTP]}{dt} = -k_{syn}[NTP][dsDNA-T7]$$

Supplementary Equation 24: Background RNA

$$\frac{d[bkRNA]}{dt} = k_{pb}^{-1}[bkRNA-P1] + k_{pb}^{-1}[bkRNA-P2] - k_{pb}[bkRNA][P1] - k_{pb}[bkRNA][P2]$$

Supplementary Equation 25: Complexed Background-P1

$$\frac{d[bkRNA - P1]}{dt} = k_{pb}[bkRNA][P1] - k_{pb}^{-1}[bkRNA - P1]$$

Supplementary Equation 26: Complexed Background-P2

$$\frac{d[bkRNA - P1]}{dt} = k_{pb}[bkRNA][P2] - k_{pb}^{-1}[bkRNA - P2]$$

Supplementary References

1. McGinnis, J. L. *et al.* In-cell SHAPE reveals that free 30S ribosome subunits are in the inactive state. *Proc. Natl. Acad. Sci. U. S. A.* **112,** 2425–2430 (2015).
